# Identification and Verification of Immune-Related Gene Prognostic Signature Based on ssGSEA for Osteosarcoma

**DOI:** 10.3389/fonc.2020.607622

**Published:** 2020-12-15

**Authors:** Bo Xiao, Liyan Liu, Aoyu Li, Cheng Xiang, Pingxiao Wang, Hui Li, Tao Xiao

**Affiliations:** ^1^Department of Orthopedics, Second Xiangya Hospital, Central South University, Changsha, China; ^2^Orthopedic Biomedical Materials Engineering Laboratory of Hunan Province, Changsha, China

**Keywords:** immune, gene, prognostic, osteosarcoma, ssGSEA, TARGET

## Abstract

Osteosarcoma is the most common malignant bone tumor in children and adolescence. Multiple immune-related genes have been reported in different cancers. The aim is to identify an immune-related gene signature for the prospective evaluation of prognosis for osteosarcoma patients. In this study, we evaluated the infiltration of immune cells in 101 osteosarcoma patients downloaded from TARGET using the ssGSEA to the RNA-sequencing of these patients, thus, high immune cell infiltration cluster, middle immune cell infiltration cluster and low immune cell infiltration cluster were generated. On the foundation of high immune cell infiltration cluster vs. low immune cell infiltration cluster and normal vs. osteosarcoma, we found 108 common differentially expressed genes which were sequentially submitted to univariate Cox and LASSO regression analysis. Furthermore, GSEA indicated some pathways with notable enrichment in the high- and low-immune cell infiltration cluster that may be helpful in understanding the potential mechanisms. Finally, we identified seven immune-related genes as prognostic signature for osteosarcoma. Kaplan-Meier analysis, ROC curve, univariate and multivariate Cox regression further confirmed that the seven immune-related genes signature was an innovative and significant prognostic factor independent of clinical features. These results of this study offer a means to predict the prognosis and survival of osteosarcoma patients with uncovered seven-gene signature as potential biomarkers.

## Introduction

Osteosarcoma is one of the most common primary malignant bone tumor and the second chief cause of cancer-related deaths in children and adolescence, accounting about 5% of childhood cancers (1, 2). Currently, the chief treatment for osteosarcoma is surgery combined with chemotherapy. Five-year survival rate of osteosarcoma patients has increase to ~70% over the last 30 years (3). However, because of drugs resistance, metastasis or recurrence, prognosis of osteosarcoma patients remains poor (2). Thus, to identify prognostic biomarkers that associate with biological heterogeneity of osteosarcoma is urgently needed to improve prognosis.

Nowadays, immune oncology has attracted much attention for its particular clinical benefits of many cancers. Immuotherapy, a novel treatment, has achieved favorable results for some cancers, such as breast cancer, hepatocellular carcinoma (4, 5). As a cellular environment, tumor microenvironment was composed of mesenchymal cells, endothelial cells, immune cells, inflammatory mediators, and extracellular matrix molecules. The bone microenvironment of osteosarcoma contains osteoblasts, osteoclasts and hematopoietic cells. Macrophages/monocytes were derived from these cells (6, 7). Multiple cytokines and growth factors were released from these cells. Immune-related genes and immune cells infiltration act as an indispensable role in tumor development and progression (8). Therefore, the comprehensive analysis of the relationship between immune-related genes and overall survival maybe provide a new reference for the treatment and prognosis of osteosarcoma.

In this study, we used ssGSEA to assign osteosarcoma patients into high-, middle- and low-immune cell infiltration clusters, and validated by ESTIMATE, CIBERSORT and K-M analysis. Then, we identified a seven immune-related genes signature correlated with the prognosis in differentially expressed genes in both osteosarcoma group and high immune cell infiltration cluster using univariate Cox and LASSO regression analysis. Finally, we assessed the accuracy of immune-related gene prognosis signature. The immune-related gene signature not only can enhance the ability to predict prognosis in osteosarcoma patients but also maybe explain the underlying mechanisms.

## Materials and Methods

### Collect and Cluster of Osteosarcoma Data

The RNA-sequencing data of osteosarcoma (n = 101) were downloaded from TARGET (Therapeutically Applicable Research to Generate Effective Treatments) (https://ocg.cancer.gov/programs/target). 95 samples of them with corresponding clinical information. The gene expression of GSE42352 were obtained from Gene Expression Omnibus (GEO) database. GSE42352 is a microarray dataset that contains 15 normal cells/tissues (12 mesenchymal stem cells, 3 osteoblast), 103 osteosarcoma tissues/cells (84 biopsies of osteosarcoma tissue, 19 cell lines). The platform of the dataset is GPL10295. We obtained 29 immune-related cells and types (9). The ssGSEA was applied to explore the different infiltration degrees of immune cell types, immune-related functions, and immune-related pathways in osteosarcoma expression profile of TARGET and GSE42352 database using the R package “GSVA”. By applying unsupervised hierarchical clustering algorithm (cutoff = 1.0), osteosarcoma samples were assigned into three clusters based on immune infiltration. Samples of osteosarcoma in the TARGET and GSE42352 were classified as high immune cell infiltration cluster, middle immune cell infiltration cluster and low immune cell infiltration cluster based on the result of ssGSEA using the R package “sparcl”. Besides, the GSE42352 was applied to analyze the differentially expressed genes between normal tissue/cells and osteosarcoma tissue/cells using the R package “limma”.

### Validation of the Effectuality of Immune Clustering

ESTIMATE was designed to count scores for reflecting the infiltration levels of immune cells and stromal cells within the tumor microenvironment on the foundation of the specific genes expression level of immune and stromal cells using the R package “ESTIMATE” (10). First, we used ESTIMATE algorithm based on the expression level of RNA-seq to count the Tumor Purity, ESTIMATE Score, Immune Score, and Stromal Score of 101 osteosarcoma samples in three clusters of TARGET database using the R package “estimate” to validate the effectuality of ssGSEA grouping and to picture clustering heatmap. The vioplots of Tumor Purity, ESTIMATE Score, Immune Score, and Stromal Score in three clusters were presented employing the R package “ggpubr”. Next, to investigate the difference of immune cell subtypes, the R package “CIBERSORT” was applied to count the proportion of 22 immune cells of all osteosarcoma samples on the foundation of expression file (11), and the difference of three clusters was validated again. Besides, we also used K-M analysis and the expression of HLA family and PD-L1 to validate the difference between three clusters applying the R package “survival” and “ggpubr” respectively.

### Distinction of Immune-Related Genes in Osteosarcoma

Based on the above-mentioned clusters, mRNA-seq expression data of TARGET and expression profiling of GSE42352 were divided into high immune cell infiltration, middle immune cell infiltration and low immune cell infiltration cluster with the same cutoff. We obtained the differentially expressed genes (|lgFC| > 1 and adj.P.Val/FDR < 0.05) between high immune cell infiltration and low immune cell infiltration cluster in two databases using the R package “edgeR”. The same criterion was applied to obtain the differentially expressed genes associated with tumorigenesis between normal tissue/cells and osteosarcoma tissue/cells using the R package “limma”. Finally, we used Venn analysis to investigate the immune-related genes from the above-mentioned results.

### GSEA Enrichment Analysis

Gene set enrichment analysis was conducted between high immune cell infiltration and low immune cell infiltration clusters of TARGET database using the R “clusterprofiler” package. The R “enrichplot” package was used to generate visual gene set enrichment maps with annotations. p < 0.05 was considered statistically significant.

### Distinction and Confirmation of Immune-Related Gene Prognostic Signature for Osteosarcoma

First, we used univariate Cox regression analysis to distinct immune-related gene in osteosarcoma samples (n = 95) with complete clinical information in TARGET dataset with significant correlation to overall survival using the R “survival” package. We then used LASSO regression analysis to screen survival related genes for significant results of univariate Cox regression analysis using the R “glmnet” package. 1000-round cross-validation for tuning parameter selection was used to prevent overfitting and the partial likelihood deviance met the minimum criteria. Finally, we constructed the prognostic signature of osteosarcoma according to the expression of immune-related genes and their relevant coefficients result from LASSO regression analysis. The formula as follows: risk score = ∑ ∑*i*coefficient(gene*i*) × expression(gene*i*). The median value of risk score was defined using the “Survminer” package, in which the osteosarcoma patients were divided into high-risk and low-risk groups. The time-dependent receiver-operating characteristic (ROC) and Kaplan-Meier (K-M) curves were used to assess the clinical prognostic capacity of the risk score using the R “timeROC”, “survival”, and “survminer” packages. Further, to assess whether risk score can be regarded as an independent predictor of overall survival of osteosarcoma patients, univariate Cox and multivariate Cox regression analysis were performed with risk score, gender, age and metastasis status as variables using the R “survival” package.

### Construction and Verification of Nomogram

The nomogram was designed to predict survival of osteosarcoma patients. Risk score, age, gender, primary tumor site and metastasis status were used to build the nomogram using R “rms” and “survival” packages. The calibration curve was used to evaluate the accuracy of the nomogram in differentiating between patient groups.

### Statistical Analysis

All statistical analyses were conducted using the R software version 4.0.1. Kaplan-Meier analysis and log-rank test were applied to assess survival and compare difference in survival between clusters as well as risk groups. p < 0.05 was regarded statistically significant.

## Results

### Construction and Validation of Osteosarcoma Clustering

**Figure 1:**
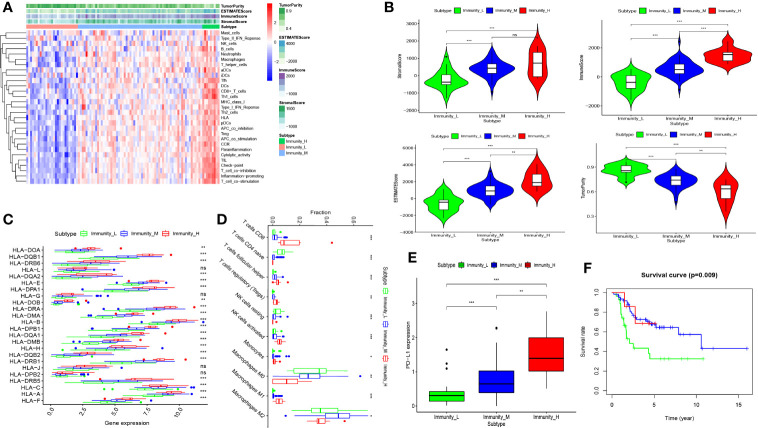
Construction and validation of osteosarcoma clustering. **(A)** The enrichment levels of 29 immune-related cells and types in the high immune cell infiltration group (Immunity_H), middle immune cell infiltration group (Immunity_M), and the low immune cell infiltration group (Immunity_L). The Tumor Purity, ESTIMATE Score, Immune Score and Stromal Score of every patient gene were showed combine with the clustering information. **(B)** The violin plot showed the difference in ESTIMATE Score, Immune Score, Stromal Score, and Tumor Purity between three clusters. **(C, D)** The expression of most HLAs and PD-L1 was a significant difference in high- (red), middle- (blue), and low- (green) immune cell infiltration cluster. **(E)** the boxplot showed the proportion of immune cells among the high- (red), middle- (blue), and low- immune cell infiltration cluster(green) using CIBERSORT algorithm. **(F)** Patients in low immune cell infiltration cluster (green) showed worse overall survival than those in (middle- (blue), and low- (green) immune cell infiltration) cluster. nap > 0.05, *p < 0.05, **p < 0.01, ***p < 0.001. ns, no statistically significant.

We obtained 101 osteosarcoma samples data from the TARGET. The ssGSEA method was applied to the RNA-sequencing data of osteosarcoma samples to assess the immune cells infiltration. Then, the richness levels of 29 immune-related cells and types in osteosarcoma samples was obtained. By applying unsupervised hierarchical clustering algorithm (cutoff = 1.0), osteosarcoma samples were assigned into three clusters based on immune infiltration, which include the high immune cell infiltration cluster (n = 9), middle immune cell infiltration cluster (n = 65), and low immune cell infiltration cluster (n = 27) (**Figure 1A** and **Supplementary Figure 1A**). To validate the practicability of the above clustering result, Stromal Score, Immune Score, ESTIMATE Score and Tumor Purity were calculated according to the expression level of osteosarcoma using the ESTIMATE algorithm. The result has shown that Stromal Score, Immune Score, and ESTIMATE Score of high immune cell infiltration cluster were higher than that of other two clusters, but Tumor Purity was opposite (**Figure 1A**). The violin plot also has shown that Stromal Score, Immune Score and ESTIMATE Score in low-, middle-, high- immune cell infiltration cluster become higher and higher, while Tumor Purity become lower and lower (p < 0.05) (**Figure 1B**). Besides, boxplot has also shown that the expression of most of HLAs and PD-L1 in low-, middle-, high- immune cell infiltration cluster become higher and higher (p < 0.01) (**Figures 1C, D**). In addition, we discovered that most of immune cells were displayed in higher proportions in high immune cell infiltration cluster using CIBERSORT algorithm (**Figure 1E**). Finally, the Kaplan-Meier (K-M) curve has shown that the osteosarcoma patients in low immune cell infiltration cluster have worse survival rate and the survival rate of three clusters have significant difference (p = 0.009) (**Figure 1F**). Obviously, all of these results demonstrate that the osteosarcoma clustering can be used for further analysis. Similarly, osteosarcoma samples in GSE42352 were also assigned into three clusters based on the richness levels of 29 immune-related cells and types, which include the high immune cell infiltration cluster (n = 44), middle immune cell infiltration cluster (n = 40), and low immune cell infiltration cluster (n = 19) *via* applying unsupervised hierarchical clustering algorithm with the same cutoff value (**Supplementary Figure 1B**).

### Identification of Differentially Expressed Genes Between Osteosarcoma and Normal Groups and Between High and Low Immune Cell Infiltration Clusters

**Figure 2:**
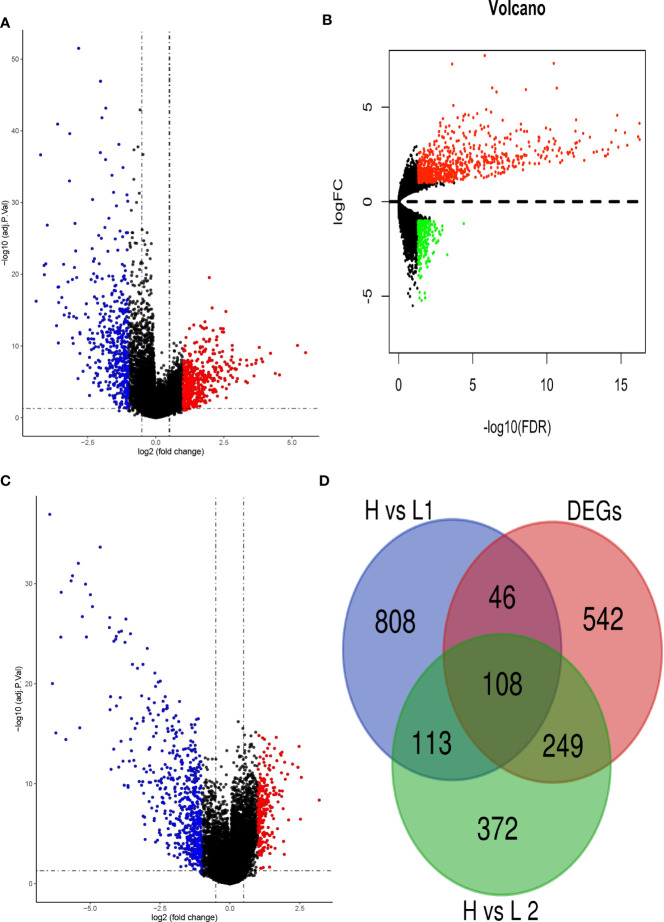
Identification of differentially expressed genes. **(A)** The volcano plot showed that 524 up-regulated genes (red) and 421 down-regulated genes (green) between osteosarcoma (T) and normal (N) tissues/cells. **(B)** the volcano plot showed that 808 up-regulated genes (red) and 267 down-regulated genes (green) between high and low immune cell infiltration cluster in TARGET dataset. **(C)** The volcano plot showed that 245 up-regulated genes (red) and 597 down-regulated genes (green) between high and low immune cell infiltration cluster in GSE42354. **(D)** Using Draw Venn Diagram to pick up the intersection, 108 differentially expressed genes were obtained.

PCA 3D plot showed the mesenchymal stem cells and osteoblast are relatively closed compared to osteosarcoma (**Supplementary Figure 2**). Using a threshold of |lgFC| > 1 and adj.P.Val/FDR < 0.05, we explored the differentially expressed genes between normal group (15 cases) and osteosarcoma group (103 cases) in the GSE42352. Then, 945 differentially expressed genes including 524 up-regulated and 421 down-regulated were obtained (**Figure 2A**). Basing on the same criterion, we obtained 1075 differentially expressed genes between high immune cell infiltration cluster and low immune cell infiltration cluster in TARGET database, with 245 up-regulated and 597 down-regulated (**Figure 2B**). Besides, 842 differentially expressed genes were obtained between high immune cell infiltration cluster and low immune cell infiltration cluster in GSE42352, with 808 up-regulated and 267 down-regulated (**Figure 2C**). Finally, through using Venn analysis of three sets of differentially expressed genes, 108 differentially expressed genes were identified in both high immune cell infiltration cluster and osteosarcoma group (**Figure 2D**).

### GSEA Enrichment Analysis

**Figure 3:**
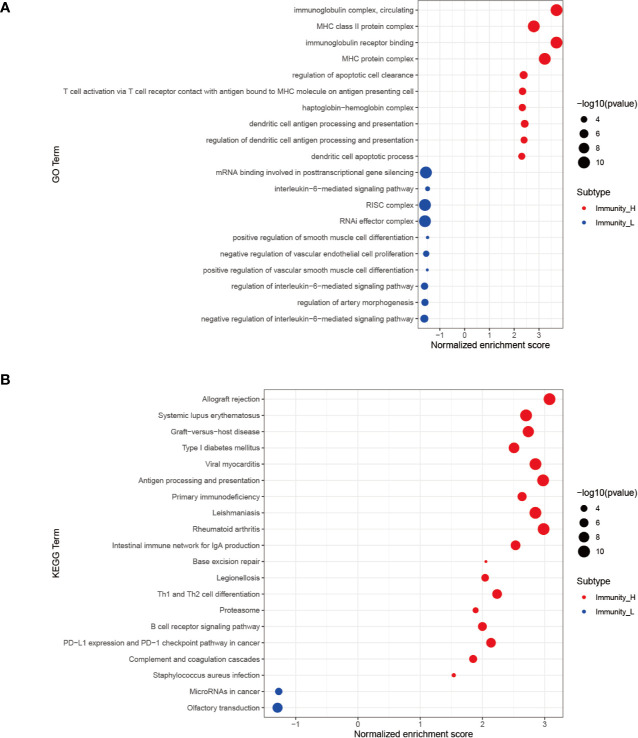
GSEA enrichment analysis. **(A)** The top 10 results of GO analysis in Immunity_H (red) and Immunity_L (green). **(B)** The results of KEGG analysis in Immunity_H (red) and Immunity_L (green).

The GO analysis of genes in the high immune cell infiltration cluster and low immune cell infiltration cluster in TARGET database has shown that these associated with MHC class II protein complex, immunoglobulin receptor binding, regulation of apoptotic cell clearance and so on (**Figure 3A**). The KEGG analysis indicated that the genes correlated with primary immunodeficiency, Intestinal immune network for IgA production, Th1 and Th2 cell differentiation, PD−L1 expression and PD−1 checkpoint pathway in cancer, B cell receptor signaling pathway and so on (**Figure 3B**).

### Distinction and Evaluation of Seven Immune-Related Genes Prognostic Signature for Osteosarcoma

**Figure 4:**
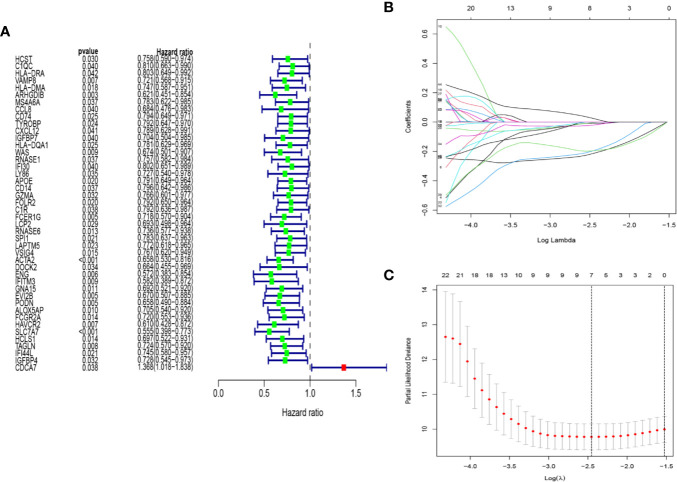
Construction of immune-related gene prognostic signature. **(A)** The p-value and HR of selected genes in univariable Cox regression analysis (p < 0.05). **(B)** The LASSO Cox analysis identified seven genes associated with prognosis. **(C)** The optimal values of the penalty parameter were defined by 1,000-round cross-validation.

We selected 95 osteosarcoma patients with complete clinical data for the further analysis. Univariate Cox regression analysis was applied to the expression profiles of 108 genes in osteosarcoma patients. From this analysis, 44 differentially expressed genes were found to have significant association with overall survival (p < 0.05) (**Figure 4A**). Then, by the LASSO regression analysis, seven genes, including IFITM3, VAMP8, ACTA2, GZMA, CDCA7, EVI2B, and SLC7A7, were identified (**Figures 4B, C**). The risk score was counted based on the expression level and its coefficient of these seven genes. Risk score = (−0.0297667 *expression of VAMP8) + (−0.0842312 * expression of GZMA) + (−0.3625101*expression of ACTA2) + (−0.3627611 * expression of IFITM3) + (0.15067589*expression of CDCA7) + (−0.0458604 * expression of EVI2B) +(−0.2311853*expression of SLC7A7).

**Figure 5:**
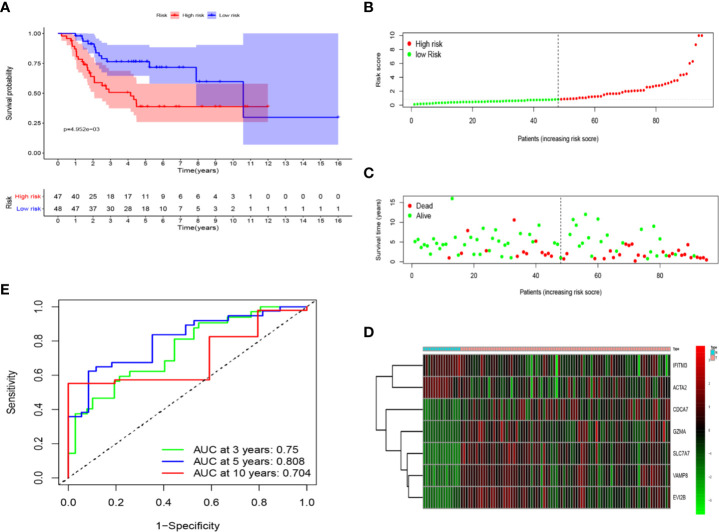
The prognostic value of seven immune-related genes. **(A)** Patients in the low-risk group (blue) showed better overall survival than those in the high-risk group (red). **(B)** The risk curve of every sample arranged by risk score. **(C)** The scatter plot of osteosarcoma samples survival overview. The red and green dots stand for death and survival, respectively. **(D)** Heatmap showed the expression level of seven genes in the normal group (blue) and osteosarcoma group (red). **(E)** The ROC curve for 3-, 5-, and 10-year overall survival of osteosarcoma patients.

We divided patients into high-risk and low-risk groups based on the median risk score. Kaplan-Meier (K-M) curves demonstrated low-risk group had a noticeably better survival than patients in high-risk group (p = 4.952e−03), showing the risk score has an effective value of prognosis (**Figure 5A**). Risk score and survival status of all osteosarcoma patients were exhibited in the risk curve and scatterplot. The mortality and risk coefficient of patients in the low-risk group were lower than that of high-risk group (**Figures 5B, C**). The heatmap of these seven immune-related genes expression level in GSE42352 indicated that five of these genes (CDCA7, GZMA, SLC7A7, VAMP8, and EVI2B) were highly expressed in the osteosarcoma group, but two of these genes (IFITM3 and ACTA2) were highly expressed in the normal group (**Figure 5D**). Besides, Time-dependent ROC curves was used to assess the precision of the seven immune-related genes signature in predicting overall survival of osteosarcoma patients at 3-, 5-, and 10- year. The area under the ROC (AUC) values of 3-, 5-, and 10- year were 0.750, 0.808, and 0.704, respectively (**Figure 5E**), showing a good capacity of our seven immune-related genes signature in predicting overall survival. As a whole, all of these analyses identify seven immune-related genes as prognostic signature for osteosarcoma.

### Assessment of Seven Immune-Related Genes Signature as Independent Prognostic Factor in Osteosarcoma Patients

**Figure 6:**
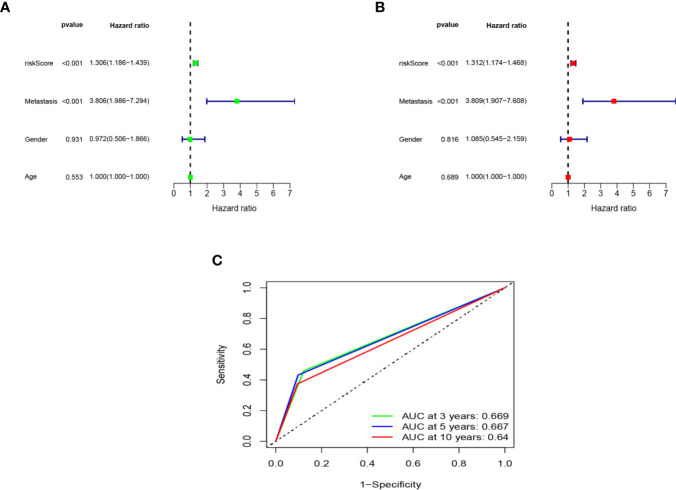
Assessment of the independent prognostic value. The univariate **(A)** and multivariate **(B)** Cox regression analysis of risk score, age, gender and metastasis. **(C)** The AUC of metastasis model based on ROC curve.

We applied univariate and multivariate Cox regression analysis to test whether the seven immune-related genes signature was an independent prognostic factor for other features, such as gender, age, and metastasis status. Both univariate and multivariate Cox regression analysis illustrated risk score and metastasis status were the independent prognostic factors (P < 0.001) (**Figures 6A, B**). Time-dependent ROC curves were used to evaluate the precision of metastasis status in predicting overall survival of osteosarcoma patients at 3-, 5-, and 10- year. The area under the ROC (AUC) values of 3-, 5-, and 10- year were 0.669, 0.667, and 0.64, respectively (**Figure 6C**) were lower than that of risk score. Both of results indicated that the seven immune-related genes signature was an independent prognostic factor in osteosarcoma patients.

### Construction and Validation of Nomogram

**Figure 7:**
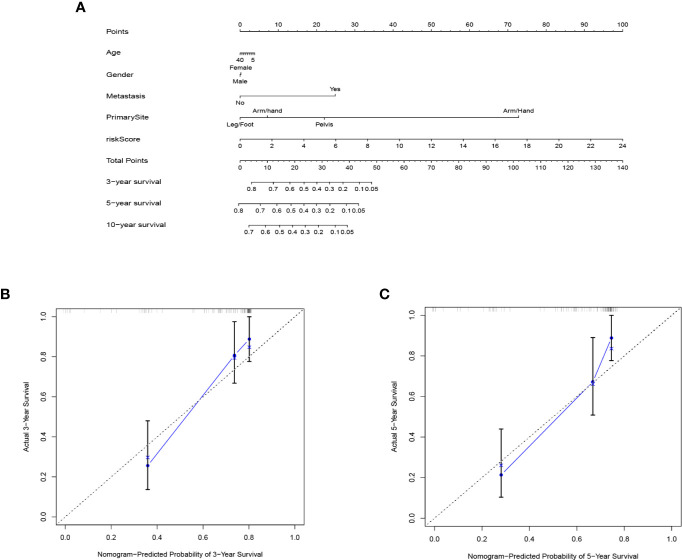
The nomogram to predict the survival rate of osteosarcoma patients. **(A)** nomogram of TARGET cohort used to predict the survival time. **(B, C)** calibration maps used to predict the 3-year **(B)** and 5-year survival **(C)**.

In order to predict the 3-year, 5-year, and 10-year overall survival of each patient, a nomogram was designed based on the TARGET. The expression signature for the seven immune-related genes, age, gender, and metastasis status were used as variables (**Figure 7A**). The calibration curve obtained compared well with the ideal model (**Figures 7B, C**).

## Discussion

Osteosarcoma derives from mesenchymal stem cells, is the most common malignant bone tumor in children and adolescence, with the highest level of heterogeneity in humans (12, 13). This heterogeneity occurs not only in the macroscopic and microscopic aspects, with heterogeneous tumor microenvironment, but in genotypes and phenotypes aspects (13). Osteosarcoma tissue is not only composed of osteoblastic, chondroblastic and/or fibroblastic but also blended with immune cells and stromal cell (14, 15). That is to say, each sample has different types of cells composition. Thus, we concentrate on the heterogeneity of osteosarcoma and correlation between tumor immune cells infiltration and tumor cells, which was of remarkable meanings to study the mechanism of tumor development and progression, and to discover new approaches of diagnosis and therapy. By using the mRNA sequencing data and clinical data of osteosarcoma obtained from TARGET, we identified and validated the seven gene prognostic signature associated with immune cell infiltration.

In this study, we divided the samples into three clusters based on the enrichment of 29 immune cell types by applying unsupervised hierarchical clustering algorithm. There were significant differences in Immune Score, Stromal Score, ESTIMATE Score and Tumor Purity between the high-, middle- and low- immune cell infiltration cluster. Then, the heterogeneity of immune microenvironment in osteosarcoma was validated by the expression level of HLAs and PD-L1 (16–18). Besides, there were also obviously difference in the proportion of most of 22 immune cells of three clusters using CIBERSORT algorithm. Finally, K-M curve indicated that the osteosarcoma patients in low immune cell infiltration cluster have lower survival rate and the survival rate of three clusters have significant difference. The seven immune-related genes signature for overall survival was constructed on the differentially expressed genes among high immune cell infiltration cluster and osteosarcoma to predict the prognosis of osteosarcoma patients. The samples were assigned into low-risk group and high-risk group based on the median risk score. K-M curve, ROC curve, and score plot and plot of survival status shown that the seven immune-related gene had a favorable predictive ability.

Five of the seven immune-related genes (CDCA7, GZMA, SLC7A7, VAMP8, and EVI2B) were highly expressed in the osteosarcoma group, while two of these immune-related genes (IFITM3 and ACTA2) were lowly expressed. Most of the seven immune-related genes have been found associate with prognosis of different types of cancers. IFITM3 enhanced the invasion and metastasis of hepatocellular carcinoma through regulating expression of MMP9 *via* the p38/MAPK pathway and promoted proliferation of hepatocellular carcinoma *via* regulating expression of c-myc through the ERK1/2 signaling pathway (19, 20). Overexpression of IFITM3 was correlated with poor prognosis and associated with some molecules of immune checkpoint and biomarkers of tumor-associated macrophage in head and neck squamous cell carcinoma (21). Previous study identified that high expression of VAMP8 was associated with oncogenesis and metastasis of osteosarcoma (22). High expression of VAMP8 enhanced the cell proliferation of glioma and glioma growth by regulating cell cycle in the G0/G1 phase (23). ACTA2 was associated with maintenance of cell shape and mechanical tension and played an important role in tumor cell migration and invasion (24). Overexpression of ACTA2 had an increased risk of distant metastasis and worse survival for lung adenocarcinoma patients (25). It has been found that immune-related gene (GZMA) is high expressed in endometrial cancer at early stage and associated with prognosis, but the mechanism of tumor progression is unclear (26). As a family member of the cell division cycle proteins, CDCA7 was markedly overexpressed in colorectal cancer and lung adenocarcinoma. CDCA7 enhance cell proliferation in lung adenocarcinoma by mediating G1 phase and regulating apoptosis. besides, colorectal cancer patients with low expression of CDCA7 had better overall survival (27, 28). It has been found that low expression of EVI2B could enhance cell proliferation, invasion and migration in colorectal cancer cells and remarkably promote tumor growth. besides, EVI2B participated in PPIs (protein-protein interactions) of T-cell-mediated system-wide modulation (29). Similarly, EVI2B was a potential prognostic biomarker for colorectal cancer (CRC) (30). SLC7A7 was a significant and independent biomarker for predicting poor prognosis and was a potential prognosis predicter of glioblastoma (31). Mounting evidence showed dysregulation of SLC7A7 was associated with resistance of chemotherapy and radiation in ovarian cancers and non-small cell lung cancer, respectively (32, 33). Studies about the tumor progression part of SLC7A7 are too limited. APOE regulates lipid homeostasis in the systemic circulation and induces inflammatory immune responses in the tumor microenvironment (34).

Then, we investigated the correlation between risk score, gender, age and metastasis status in patients with osteosarcoma by univariate Cox and multivariate Cox regression analysis. Both of results showed that risk score and metastasis status maybe the independent prognostic factors in osteosarcoma patients (p < 0.001). To find out which of the two independent prognostic factors can better predict overall survival in osteosarcoma patients, we constructed a metastasis status model. The AUC values of ROC curve were applied to assess the accuracy of the model. The AUC values of 3-, 5-, and 10- year are obviously lower than those of the immune-related gene signature, suggesting that risk score is an independent prognostic factor and the predictive ability is significantly better than that of metastasis status.

Finally, we designed a nomogram to predict prognosis of osteosarcoma patients based on the risk score of seven immune-related gene signature, age, gender, metastasis status and primary tumor site. From this nomogram we were able to predict the 3-, 5-, and 10-year survival of osteosarcoma. Consistently, the plots of calibration demonstrated that the signature could accurately evaluate the survival of osteosarcoma patients. Although, our prognostic signature had a favorable predictive capability, the prognostic model can be optimized by consolidating with other independent datasets or even improved by optimizing the LASSO results in the future.

Some limitations of our study should be admitted. First, lack of sufficient samples and some other clinical pathological features. Second, the performance of our prognostic signature should be validated in more osteosarcoma datasets. Finally, all the results were based on public datasets and should be further confirmed by actual experiments.

In conclusion, the seven immune-related genes signature identified as the independent prognostic significance for osteosarcoma. These results of this study offer a means to predict the prognosis and survival of osteosarcoma patients and maybe provide promising targets for immunotherapy.

## Data Availability Statement

The original contributions presented in the study are included in the article/**Supplementary Material**. Further inquiries can be directed to the corresponding authors.

## Author Contributions

BX had the idea, collected data and wrote the first draft, HL, LL and AL did statistical analysis, BX, CX, and PW created the figures, HL and TX drafted the final manuscript. All authors contributed to the article and approved the submitted version.

## Funding

This work was supported by Key Research and Development Program of Hunan Province 2017GK2120 (to TX).

## Conflict of Interest

The authors declare that the research was conducted in the absence of any commercial or financial relationships that could be construed as a potential conflict of interest.
