# Supplementary material for: Identification and Verification of Immune-Related Gene Prognostic Signature Based on ssGSEA for Osteosarcoma
Source: Front Oncol. 2020 Dec 15;10:607622. doi: 10.3389/fonc.2020.607622 (PMC7771722; doi:10.3389/fonc.2020.607622)
Supplement: Supplementary file 1 [file DataSheet_1.docx]

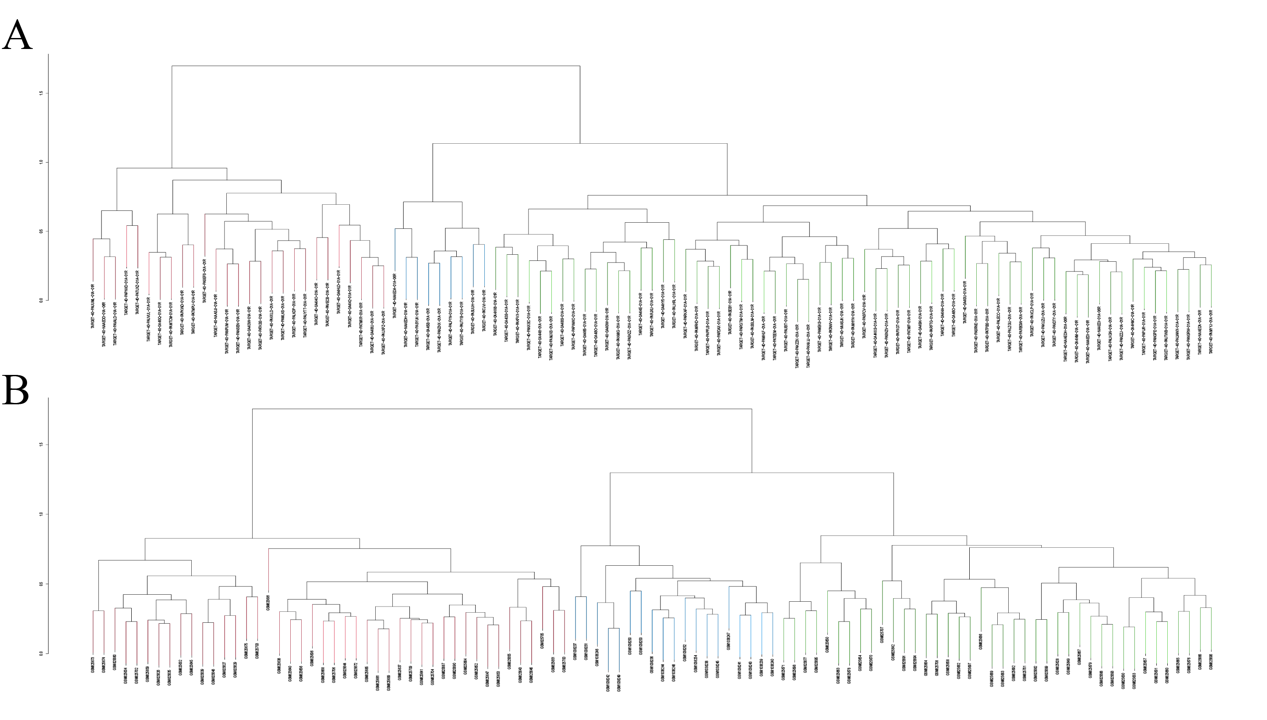


Figure.S1: Cluster dendrogram of osteosarcoma samples in the TARGET (A) and GSE42352 (B) datasets.


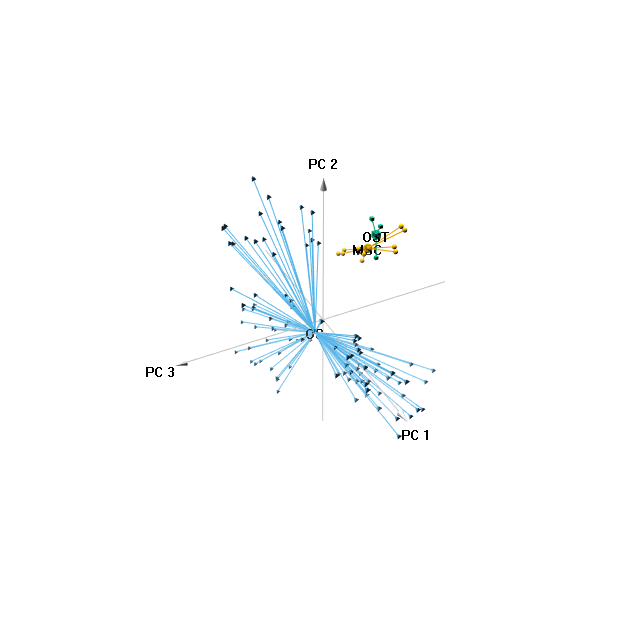


Figure.S2: PCA 3D plot of mesenchymal stem cells (MSC) (yellow), osteoblast (OST) (green) and osteosarcoma (OS) (blue) in GSE42352.
